# Theta-burst rTMS in schizophrenia to ameliorate negative and cognitive symptoms: study protocol for a double-blind, sham-controlled, randomized clinical trial

**DOI:** 10.1186/s13063-024-08106-9

**Published:** 2024-04-17

**Authors:** Gábor Csukly, Boglárka Orbán-Szigeti, Karolin Suri, Réka Zsigmond, Levente Hermán, Viktória Simon, Anita Kabaji, Barnabás Bata, Péter Hársfalvi, Edit Vass, Éva Csibri, Kinga Farkas, János Réthelyi

**Affiliations:** 1 Department of Psychiatry and Psychotherapy, Semmelweis University, Balassa 6, Budapest, 1083 Hungary; 2 Department of Cognitive Science, Faculty of Natural Sciences, Budapest University of Technology and Economics, Budapest, Hungary; 3 Department of Biostatistics, University of Veterinary Medicine Budapest, Budapest, Hungary; 4 BiTrial Clinical Research, Budapest, Hungary

**Keywords:** Schizophrenia, Theta-burst, Transcranial magnetic stimulation, Negative symptoms, Cognitive symptoms

## Abstract

**Background:**

Treatment effects of conventional approaches with antipsychotics or psychosocial interventions are limited when it comes to reducing negative and cognitive symptoms in schizophrenia. While there is emerging clinical evidence that new, augmented protocols based on theta-burst stimulation can increase rTMS efficacy dramatically in depression, data on similar augmented therapies are limited in schizophrenia. The different patterns of network impairments in subjects may underlie that some but not all patients responded to given stimulation locations.

**Methods:**

Therefore, we propose an augmented theta-burst stimulation protocol in schizophrenia by stimulating both locations connected to negative symptoms: (1) the left dorsolateral prefrontal cortex (DLPFC), and (2) the vermis of the cerebellum. Ninety subjects with schizophrenia presenting negative symptoms and aging between 18 and 55 years will be randomized to active and sham stimulation in a 1:1 ratio. The TBS parameters we adopted follow the standard TBS protocols, with 3-pulse 50-Hz bursts given every 200 ms (at 5 Hz) and an intensity of 100% active motor threshold. We plan to deliver 1800 stimuli to the left DLPFC and 1800 stimuli to the vermis daily in two 9.5-min blocks for 4 weeks. The primary endpoint is the change in negative symptom severity measured by the Positive and Negative Syndrome Scale (PANSS). Secondary efficacy endpoints are changes in cognitive flexibility, executive functioning, short-term memory, social cognition, and facial emotion recognition. The difference between study groups will be analyzed by a linear mixed model analysis with the difference relative to baseline in efficacy variables as the dependent variable and treatment group, visit, and treatment-by-visit interaction as independent variables. The safety outcome is the number of serious adverse events.

**Discussion:**

This is a double-blind, sham-controlled, randomized medical device study to assess the efficacy and safety of an augmented theta-burst rTMS treatment in schizophrenia. We hypothesize that social cognition and negative symptoms of patients on active therapy will improve significantly compared to patients on sham treatment.

**Trial registration:**

The study protocol is registered at “ClinicalTrials.gov” with the following ID: NCT05100888. All items from the World Health Organization Trial Registration Data Set are registered. Initial release: 10/19/2021.

**Supplementary Information:**

The online version contains supplementary material available at 10.1186/s13063-024-08106-9.

## Background

Schizophrenia is a major mental disorder that affects approximately 1% of the population worldwide. Social cognition impairments and negative symptoms such as blunted affect or emotional withdrawal strongly contribute to the psychosocial functioning deficits and long-term disability in schizophrenia [1]. The state-like and trait-like components of social cognition are impaired in schizophrenia [2, 3].

The effect of current antipsychotic medications on social cognition and negative symptoms is strongly limited [4]. Therefore, the significance of non-pharmacological treatments in schizophrenia, such as rTMS, is emerging. Transcranial magnetic stimulation (TMS) is a noninvasive form of brain stimulation in which a changing magnetic field is used to cause an electric current at a specific brain area through electromagnetic induction. While evidence for the efficacy of rTMS treatment in depression is solid and led to FDA approval in 2008, findings on rTMS treatment in schizophrenia are somewhat controversial. Some results show improvement, especially in facial expression recognition and social cognition applying a standard high-frequency (10 Hz) stimulation protocol on the left DLPFC [5]. Some other approaches trying to decrease negative symptoms were unsuccessful [6]. Similarly, the results of other studies applying the same standard 10 Hz stimulation protocol on the temporoparietal cortex to ameliorate auditory hallucinations were also contradictory since some studies showed positive results [7], while others failed to find evidence compared to sham stimulation [8].

Studies applying rTMS to improve negative symptoms in schizophrenia have typically targeted the dorsolateral prefrontal cortex (DLPFC), which is based on neuroimaging findings of reduced DLPFC activation in patients with negative symptoms (e.g., [9]) and emotion processing. A meta-analysis found that rTMS stimulation of the left DLPFC is more effective than sham in treating negative symptoms of schizophrenia. However, the evidence was found to be low quality [10]. The mean weighted effect size compared to sham stimulation was 0.64 (0.32–0.96; *k* = 22, total *N* = 827). Studies with younger participants showed more substantial effects than those with older participants.

The recognition of the role of the cerebellum in schizophrenia pathology termed “cognitive dysmetria” is not new [11]. Furthermore, there is emerging evidence that the brain network consists of the DLPFC, and the vermis of the cerebellum is impaired in schizophrenia. The stimulation of the vermis can lead to the amelioration of negative symptoms [12, 13]. In a recent study, Brady et al. [12] stimulated the cerebellum of patients in a combined TMS-fMRI study. They showed that the improvement in DLPFC-Cerebellar functional connectivity was strongly correlated with the decrease in PANSS negative symptom severity.

Besides the stimulation location, the number of (daily) delivered pulses can also be the key to efficacy. A recent study showed that 61% of non-responders to repetitive transcranial magnetic stimulation (rTMS) responded with further treatment [14], suggesting that FDA-approved protocols may be underdosing. In previous research, 1,800 pulses have produced long-lasting changes in cortical excitability (15) and optimally produced the intended cellular changes [15]. There is also emerging clinical evidence that new, augmented protocols based on theta-burst stimulation can increase rTMS efficacy dramatically in depression. These protocols almost double the response and remission rates in depression by using intermittent theta-burst stimulation (3 pulses of stimulation are given at 50 Hz, repeated every 200 ms, and a 2-s train of TBS is repeated every 10 s). Still, delivering 1200, 2400, or even 18,000 stimuli daily compared to the standard 600 stimuli [16, 17]. Such augmented protocols have not been tested in schizophrenia yet.

Treatment effects of conventional approaches with antipsychotics, other pharmacological agents, or psychosocial interventions are limited and not clinically significant in reducing negative symptoms and improving social outcomes [4]. Based on the literature review, we can summarize that rTMS, and specifically, theta-burst rTMS (TB-rTMS), can be an excellent opportunity to enhance emotion processing and ameliorate negative symptoms in schizophrenia. However, optimal protocols are to be found. Based on recent genetic findings, it seems that schizophrenia is not a single disease but multiple genetically distinct disorders, in other words, a group of heritable disorders caused by a moderate number of separate genotypic networks associated with several distinct clinical syndromes [18]. Accordingly, several different brain networks (e.g., default mode network, cortico-cerebellar-thalamic-cortical circuit) are impaired in schizophrenia, while none are impaired in all patients [19, 20]. This different pattern of network impairments in subjects may underlie that some but not all patients responded to a given stimulation location. Taken together these evidences, we propose an augmented theta-burst stimulation protocol in schizophrenia by stimulating both locations connected to negative symptoms, namely the vermis of the cerebellum and the left DLPFC. While the temporoparietal area can also be considered a potential stimulation target, we excluded this target since this location is primarily connected to positive symptoms [21], and we intend to focus on negative symptoms. Besides multi-location stimulation, based on the recent findings on augmented protocols, we intend to deliver 1800 pulses daily to each location, which is 3600 pulses daily compared to the 600 pulses of standard theta-burst protocols. To our knowledge, this would be the first study applying multi-location (DLPFC and cerebellum) augmented theta-burst stimulation in schizophrenia.

The major target of the study is to confirm the safety and efficacy of our augmented protocol of theta-burst TMS in schizophrenia. We aim to confirm the beneficial effects of rTMS treatment on multiple aspects of the disorder such as (1) clinical aspect in terms of PANSS negative score, (2) social cognition such as Theory of Mind (ToM), (3) neurocognition such as cognitive flexibility, and (3) safety in terms of detected serious adverse events (SAEs).

Negative symptom severity correlates closely with functional outcome, and these symptoms respond the least to antipsychotic medication. Therefore, the need for new effective treatments is extremely important. Based on recent meta-analyses [10, 22], we hypothesize that decrease in PANSS negative score will be significantly larger in the active stimulation group compared to the sham group.

Social cognition impairment is a key domain in schizophrenia related to negative symptoms and affects daily functioning and quality of life [2, 23, 24]. The “Reading the Mind in the Eyes Test (RMET)” and the faux-pas test are sensitive measures of theory of mind and social cognition in schizophrenia. We expect a significantly larger improvement in the active stimulation group relative to the sham group in the RMET total score and in the correct answers in the Faux pas test.

Cognitive flexibility is severely impaired in schizophrenia, and the Wisconsin Card Sorting Test (WCST) is a widely used measure of it. There is strong evidence that cognitive flexibility measured by WCST is correlated with negative symptom severity [25] in schizophrenia. Therefore, we plan to compare the change in WCST performance regarding perseverative errors between study groups. We expect a larger improvement of cognitive flexibility in the active stimulation group. Furthermore, a computational model of the WCST will be used, and the following reinforcement parameters will be estimated: R (reward sensitivity), P (punishment sensitivity), and D (choice consistency) [26, 27].

Several previous investigations in depression [28] and schizophrenia [29] showed that theta-burst rTMS is safe and well-tolerated. However, this will be the first study with multiple location (vermis and l-DLPFC) stimulation. Therefore, it is crucial to monitor adverse events and confirm the safety of this new procedure. Based on previous studies, the most common side effect is a mild headache, while the most serious is an epileptic seizure. However, the latter is very uncommon (1/10,000 treatment sessions). Our hypothesis regarding safety is that there will be no difference between study groups in terms of serious adverse events (SAEs). In case of a serious adverse event, patient will discontinue the treatment and will be unblinded.

## Methods

This protocol is compliant with the SPIRIT 2013 guideline for study protocols [30–32], the SPIRIT 2013 checklist is in Additional file 1: Table S1.

### Design, subjects, and power analysis

We are planning a double-blind sham stimulation-controlled study with two randomized study groups. Since there were previous studies with theta-burst stimulation in schizophrenia, this is a phase II medical device study to evaluate the clinical performance of the theta-burst rTMS protocol in schizophrenia. This superiority study will compare clinical performance in terms of efficacy and safety to sham stimulation. Ninety patients meeting the DSM-V [33] criteria for schizophrenia will be enrolled in the study. Based on a recent meta-analysis on rTMS of the frontal cortex for improving negative symptoms [10], we assumed an effect size of 0.64. Using this assumption and incorporating the baseline value as a covariate, we calculated that the probability is equal to or greater than 90% (beta = 0.90) to find a significant (alpha = 0.05) difference between study groups in negative symptom improvement with this sample size (SAS PROC GLMPOWER). Patients will be assigned to the active and the sham group in a 1:1 ratio by an adaptive randomization algorithm implemented in R [34]. The algorithm took age, sex, education, and negative PANSS score into account. Patient enrolled into the study will receive sequential patient codes, while treatment (active vs. sham) will be assigned to these codes. Patients from the outpatient care of the Department of Psychiatry and Psychotherapy, Semmelweis University will be enrolled who met all the inclusion and none of the exclusion criteria. An enrollment period of 30 months is planned, therefore approximately three subjects need to be included per month to reach the target sample size. Based on the patient flow in the outpatient unit we can screen 4–5 subjects and enroll three subjects monthly. Inclusion criteria are (1) diagnosis of schizophrenia or schizoaffective disorder; (2) clinically stabilized on antipsychotic: a stable dose of antipsychotic medication for > 4 weeks; (3) age 18–55 years, and (4) presence of negative symptoms (based on PANSS): a negative subscore ≥ 16 points and one of items N1–N7 scoring ≥ 4 or two items N1–N7 scoring ≥ 3. The exclusion criteria are (1) any significant neurological illness; (2) intellectual disability; (3) history of head injury with loss of consciousness for more than 1 h; (4) history of epileptic seizures or epileptic activity on the baseline EEG (evaluated by an expert in clinical EEG and epilepsy); (5) alcohol or drug abuse within the past 3 months; (6) depressive episode or antidepressant treatment in the past 4 weeks; (7) ECT in the medical history; (8) implanted pacemaker, implanted drug pump, cochlear implant, implanted defibrillator, implanted neurostimulator or any other TMS incompatible implanted metal device; (9) skin surface is severely injured in the stimulated region of the skull; (10) sclerosis multiplex; (11) pregnancy; (12) severe sleep deprivation; (13) severe heart failure; (14) increased intracranial pressure; (15) intreated migraine; and (16) severe positive symptoms interfere with cognitive tests.

**Fig. 1:**
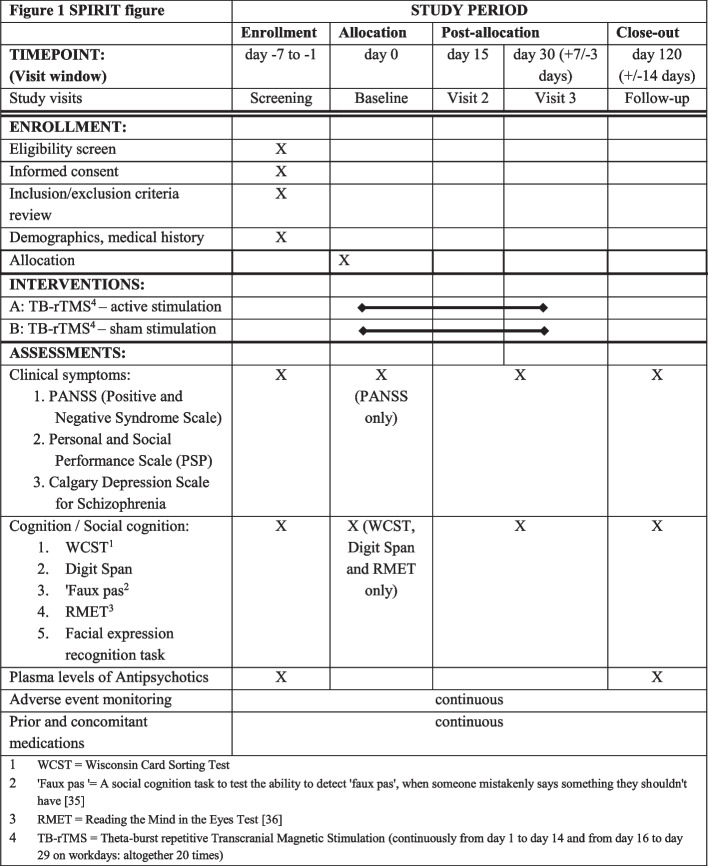
SPIRIT figure

Cognitive assessments and PANSS rating by an independent (blind to treatment condition) rater will be performed on all three visits: baseline, visit 2 (day 15), and visit 3 (day 30). A follow-up visit will be scheduled 3 months after visit 3 to assess PANSS and cognitive test battery (Fig. 1).

### TMS treatment protocol

The TBS sessions will be delivered using the Magstim Rapid2 Plus1 stimulator (Magstim Company, Ltd). The TBS parameters we adopted follow the standard TBS protocols, with 3-pulse 50-Hz bursts given every 200 ms (at 5 Hz) and an intensity of 100% active motor threshold, as measured from the right first dorsal interosseous muscle by a 700-mm figure-of-eight coil [16, 37]. An identical-looking D70 Air Film Sham Coil will be used for sham stimulation. The sham coil also generates a magnetic field, that can be sensed by the participants, but this field does not penetrate the skull. We plan to deliver 1800 stimuli to the vermis and 1800 stimuli to the left DLPFC daily in two 9.5-min-long blocks (separated by 50 min inter stimulation intervals (ISI)) for 4 weeks (altogether 20 × 2 × 1800 = 72,000 stimuli) with a 100% motor threshold [17]. We do not intend to apply strategies to improve adherence.

### Procedures to improve the blinding process

Patients, care providers, and raters will be blinded to treatment assignment, only study nurses, who generate the treatment allocation and deliver the pulses will be unblinded. Treatment allocation is generated by the aforementioned R algorithm (see the “Methods” section for further details) on a computer operated by the study nurses. The computer is password-protected and used by the study nurses exclusively. Study nurses do not participate in any other activities in the study such as efficacy assessments. All patients will be instructed that they will be treated with TBS but will be blind to the individual group assignment. The study nurse who will deliver TBS will not take part in any assessments. All efficacy outcome measures will be assessed by blinded study personnel (raters), who will not be permitted access to the treatment sessions. Patients will be instructed not to disclose any details of the treatment session with the raters, and a research assistant will monitor the whole rating period to ensure that the procedure will be blinded. We will question all patients about the group assignment at visit 3 (end of treatment visit).

### Clinical measures

The Positive and Negative Syndrome Scale (PANSS) will be administered to all patients to assess positive, negative, and general symptom severity. Everyday functioning will be measured by the Personal and Social Performance Scale (PSP) [38], while depressive symptoms will be assessed by the Calgary Depression Scale for Schizophrenia [39, 40]. Furthermore, the following demographic data will be collected from all participants: age, gender, education, job status, accommodation, family status, medications, smoking status, illness duration, schizophrenia subtype, and handedness. Since patients will be on antipsychotic medication, the mean chlorpromazine equivalent dose will be calculated [41].

### Measures of cognition and social cognition

The following tests will be performed to assess cognitive and social cognitive functioning: (1) Reading the Mind in the Eyes Test (RMET) [36]; (2) “Faux pas” test [35]; (3) Wisconsin Card Sorting Test (WCST); (4) Digit Span Forward and Backward tests; and (5) Karolinska Directed Emotional Face set of facial emotion recogniton.

In order to assess the capacity of mental state discrimination, the Revised Version of the RMET will be used [36]. The RMET presents participants with 36 black-and-white photographs of the eye region of the face, one at a time. Each photo shows the eye region of a different actor or actress. Pictures are of equal size and depict an equal number of male and female faces. Participants will be asked to choose which of four words (one target and three foils), displayed on the screen, best describes the mental state of the actor/actress. Although the RMET seems to be an emotion recognition paradigm, results from functional neuroimaging studies revealed test-related activation in brain areas related to ToM (dorsomedial prefrontal cortex and superior temporal cortex) [42]. This unique feature of the task may be due to direct instructions to attribute mental states, the application of complex social emotions, and only the presence of eye regions. The latter two factors lead to considerable ambiguity of social information that can be solved by active mentalization. A further advantage of the RMET is that numerous schizophrenia studies have applied this measure so far and confirmed that RMET is a reliable tool to detect differences in ToM between patients with schizophrenia and healthy controls [2, 23].

The Faux-pas test assesses the ability to recognize “Faux pas”: someone mistakenly saying something they should not have [35]. The test is considered an advanced test of Theory of Mind ability as it requires subtle social reasoning: one must be able to appreciate that two protagonists might have different knowledge states and also the emotional impact the statement can have on the listener. It is a well-known instrument used to evaluate theory of mind (ToM) in autism spectrum disorders or schizophrenia. The test includes 20 short stories containing incidents of faux pas. Each story is read to the individual, who is then asked questions to determine whether or not they recognized the faux pas. Understanding the mental states behind the kinds of actions presented in the Faux Pas task can be broken down into several distinct subtasks to make clear where the respondent is having trouble. The subject gets one point for each question answered correctly. As a result, the proportion of correct answers is calculated, where the maximum score ratio is 1.0. The test indicates a deficit below 0.75.

The WCST is a broadly used tool to measure executive functioning, such as concept formation, set-shifting, and flexibility [43]. In this study, a computerized, 100-card version will be used. The number of perseverative errors is the major outcome variable of the test, its minimum is 0, while it has no theoretical maximum value. Higher values indicate worse outcomes. Punishment sensitivity (P) from the reinforcement learning model of WCST will also be used as an outcome variable [26]. Its minimum value is 0 and it has no theoretical maximum value. Lower values indicate worse outcomes.

The Digit Span Forward and Backward tests require subjects to remember and rearrange short lists of numbers. These tests assess short-term memory span [43]. The outcome variable is the number of items (numbers) the participant can recall, and lower values indicate a worse outcome.

The Karolinska Directed Emotional Face set is an emotion recognition task [44], where subjects have to identify emotional expressions from photographs of 8 male and 8 female subjects. The pictures are chosen from the Karolinska Directed Emotional Face set. There are 3 photographs of each face (happy, neutral, and sad). Hit rate is the primary outcome measure, which ranges from 0 to 100%, and lower values indicate worse outcomes.

### Laboratory

Plasma levels of antipsychotic medications and their metabolites will be measured during enrollment and close-out by the Department of Laboratory Medicine (Semmelweis University) as part of routine clinical care. Plasma levels of antipsychotics and their metabolites will be measured by liquid chromatography–tandem mass spectrometry (LC–MS-MS). Samples will be stored only for 2 weeks, then they will be destroyed. All these procedures are part of routine clinical care.

### Statistical analysis plan

The primary endpoint will be the difference in negative symptom score (sum of PANSS items N1–N7) from baseline. Restricting the analysis to the participants with complete data would lead to biased and inefficient estimates. Data imputation methods and Maximum Likelihood Estimation (MLE) are two widely accepted approaches in clinical trials, however, MLE is easier to apply. Linear Mixed Model (LMM) analysis applying maximum likelihood estimation is a good alternative to data imputations in handling missing data. Therefore, the difference between study groups (active and sham) will be analyzed by a Linear Mixed Model analysis (PROC MIXED in SAS) with the difference (relative to baseline) in negative symptom score as the dependent variable and treatment group, time (visit), and treatment-by-visit interaction as predicting (independent) variables, while baseline negative symptom score will serve as covariate [45]. An unstructured covariance matrix will be used to model within-subject effects. If the model fails to converge using the unstructured covariance matrix, the following covariance structures will be modeled in the order given: heterogeneous Toeplitz, heterogeneous compound symmetry, heterogeneous autoregressive (1), Toeplitz, compound symmetry, autoregressive (1), variance components. The first covariance structure that allows for convergence will be selected for the final model. The effects of the same predictor variables on cognitive and social cognitive outcome variables will be analyzed in the same mixed model (separately for all cognitive and social cognitive tests).

Summary statistics for the PANSS negative score (observed and change from baseline) will be presented for all visits from baseline through visit 3. For change from baseline values at each post-baseline visit, LS means, and standard errors (SE), the between-group difference in LS means with the corresponding 95% confidence interval, *p*-value, and effect size will also be presented. In addition, LS mean ± SE over time for the change from baseline values by treatment group will also be presented in line plots.

No interim analysis are planned for this investigation.

### Analysis sets

The following analysis sets will be used:

#### Randomized analysis set

The randomized analysis set will consist of all unique subjects who were randomized.

#### Safety analysis set

The safety analysis set will consist of a subset of subjects in the randomized analysis set who received at least one theta-burst stimulation in one location.

#### Full efficacy analysis set

The full analysis set will consist of a subset of subjects in the safety analysis set who have both a baseline value and at least one post-baseline value for the PANSS negative score.

Subjects will be classified according to the randomized treatment assignment.

#### Per-protocol analysis set

The per-protocol analysis set will consist of a subset of subjects in the full efficacy analysis set who are at least 80% compliant (received 80% of the planned stimulation) and do not have any protocol deviations, which is considered to have a substantial impact on primary efficacy outcome. Before the clinical database lock, the precise reasons for excluding subjects from the Per-protocol analysis set will be fully defined and documented a priori.

### Patient and public involvement statement

Patients or the public were not involved in the design, conduct, reporting, or dissemination plans of our research.

### Prohibited concomitant medications

If any antidepressant medication or a new antipsychotic medication should be given to a subject, the patient should be withdrawn from treatment.

### Data management and data monitoring

Personal data of participants and data concerning their health and possible illness will be used by the investigators for the administration, conduct, scientific, and statistical analysis of the study, taking into account the Data Protection Act, which of course implies that their name will not appear anywhere. They also take responsibility for ensuring that any personal data do not fall into the hands of unauthorized persons. Clinical variables will be analyzed in a reversible anonymized format using codes. Access to the codes is restricted to the investigators and study nurses.

Clinical data will be collected utilizing OpenClinica Community Version: 3.12.2. OpenClinica EDC (hereinafter referred to as Openclinica), an Electronic Data Capture (EDC) Clinical Data Management system developed by Akaza, Inc. for collecting eCRFs. OpenClinica is an open-source EDC system that is compliant with 21 CFR Part 11, GCP, and HIPAA. User access to the application will be implemented via a secure internet connection. The application interface will be accessible through a URL. The EDC system is hosted by Semmelweis University, Department of Psychiatry and Psychotherapy. All users are required to complete EDC training related to their project role. After training each user will sign and return the meeting training record to document the completion of their training and OpenClinica access will subsequently be granted via e-mail.

The trial will be conducted according to the Good Clinical Practice (GCP) guidelines, and study monitoring will be conducted by an independent CRO (Contract Research Organization) in accordance with the study-specific Monitoring Plan (MP). 100% Source Data Verification (SDV) will be performed to verify that the reported trial data are accurate and complete. No study audits are planned for this investigation based on the risk assessment performed on the trial.

Given the low risks of the intervention, the fact that similar stimulation protocols have been applied in previous studies and the unlikelihood of critical safety concerns directly related to implementing the intervention, the trial will not have a data monitoring committee nor interim analysis stopping rule.

### Withdrawal of patient from study

Patients may withdraw from the study at any time and for any reason without prejudice to their future medical care by the investigator or at the study site. Every effort should be made to keep patients in the study. The reasons for patients not completing treatment and/or the reasons for patients not completing the study will be recorded. A patient may be withdrawn from the study for any of the following reasons:

1. Noncompliance with the protocol or significant protocol violation.
2. A serious or intolerable AE(s)
3. Lost to follow-up.
4. The patient withdraws consent.
5. New antipsychotic or antidepressant medications have to be given during the study

A worsening of the disease does not in itself imply a withdrawal, however, hospitalization due to worsening of symptoms (i.e., psychotic episode) is an SAE that implies a dropout. If a patient reports any intolerable AE, there is no possibility to decrease stimulation intensity, in such cases patient must be withdrawn. The need for a new antipsychotic or antidepressant medication also implies a dropout.

When a patient withdraws from active participation in the study, the reason(s) for discontinuation shall be recorded by the investigator on the relevant page of the eCRF. Whenever possible, all patients who discontinue treatment or withdraw from the study prematurely will undergo all assessments at the early withdrawal visit. Patients who fail to return for final assessments will be contacted by the site in an attempt to collect final data. The investigator should show due diligence and explore all possible options to reach a patient who fails to attend a visit. The investigator must document all attempts to contact the patient in the medical records/source documents (at least 3 documented approaches, via phone, e-mail, or regular mail). It is vital to obtain follow-up data on any patient withdrawn because of an AE. In the event that a patient has to be withdrawn from the study due to a serious adverse event, the patient should be followed until the condition is stabilized or the event is no longer considered clinically significant. In every case, efforts must be made to undertake protocol-specified, safety, and follow-up procedures. If patients are unable or unwilling to return for this follow-up visit, the site will document their efforts to bring the patients in through two documented telephone calls and a registered letter.

### Adverse event reporting

Reports of adverse events, accidents, serious and unexpected adverse reactions, and device malfunctions will be sent immediately to “National Institute of Pharmacy and Nutrition” at “amd.vig@ogyei.gov.hu” (with the file number of the decision authorizing the clinical trial).

### Communication of the results

After completion of the study, the data will be considered for reporting for publication in a scientific peer-reviewed journal. The principal investigator will be responsible for this activity and will work with the investigators to determine how the manuscript is written and edited, the number and order of authors, the publication to which it will be submitted, and other related issues. The principal investigator has final approval authority over all such issues.

### Insurance

All subjects who participated in the study will be insured in accordance with Hungarian legislation for the study-related activities, i.e., for TMS or blood taking.

## Discussion

**Fig. 2:**
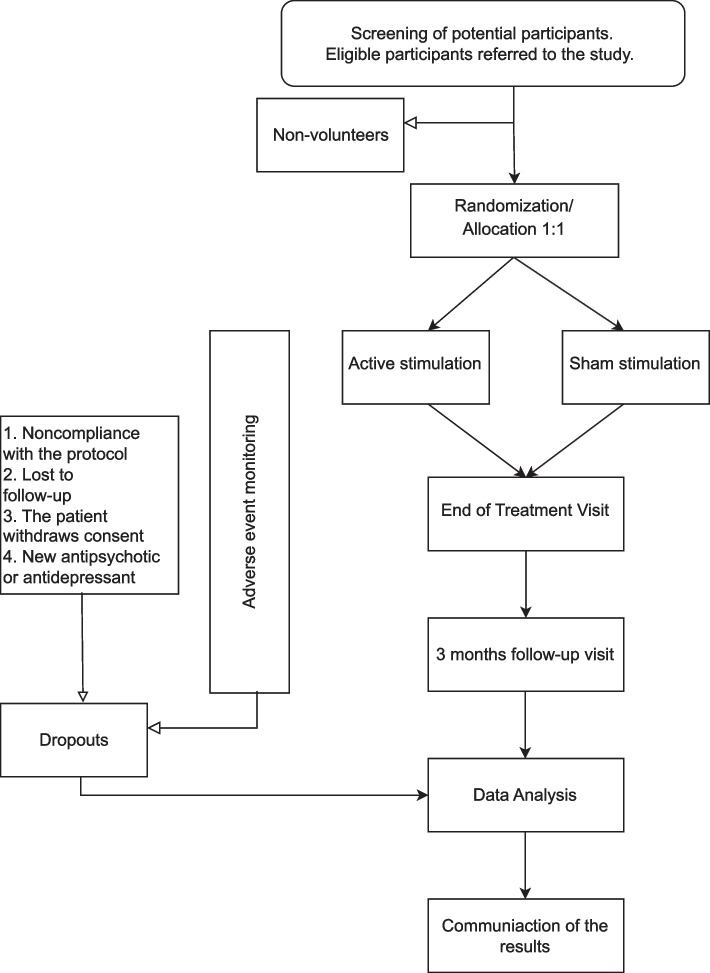
Flowchart

This is a double-blind, sham-controlled, randomized medical device study to assess the efficacy and safety of an augmented theta-burst rTMS treatment in schizophrenia (Fig. 2). We hypothesize that social cognition, executive functioning, and negative symptoms of patients on active therapy will improve significantly compared to patients on sham treatment.

## Trial status

The protocol version is 1.0 (21/03/2023). We started recruitment in late 2022, and the expected end of data collection is by the end of 2024.

### Supplementary Information

**Supplementary Material 1.**

## Data Availability

The study protocol is registered at 'clinicaltrials.gov 'with the following ID: NCT05100888. Initial release: 10/19/2021. All items from the World Health Organization Trial Registration Data Set are registered. All investigators will be given access to the cleaned data sets. Project data sets will be housed on the file transfer protocol site created for the study, and all data sets will be password protected. The raw data will also be available at “ClinicalTrials.gov.”

## Abbreviations

AE: Adverse event
CRO: Contract Research Organization
DLPFC: Dorsolateral prefrontal cortex
EDC: Electronic Data Capture
FDA: Food and Drug Administration
GCP: Good Clinical Practice
HIPAA: Health Insurance Portability and Accountability Act
ISI: Inter stimulus interval
LMM: Linear mixed model
MLE: Maximum Likelihood Estimation
MP: Monitoring Plan
PANSS: Positive and Negative Syndrome Score
PSP: Personal and Social Performance Scale
RMET: Reading the Mind in the Eyes Test
SAE: Serious adverse event
SDV: Source Data Verification
SE: Standard error
TMS: Transcranial magnetic stimulation
TBS: Theta-burst stimulation
TB-rTMS: Theta-burst repetitive transcranial magnetic stimulation
ToM: Theory of Mind
WCST: Wisconsin Card Sorting Test
